# Folding of xylan onto cellulose fibrils in plant cell walls revealed by solid-state NMR

**DOI:** 10.1038/ncomms13902

**Published:** 2016-12-21

**Authors:** Thomas J. Simmons, Jenny C. Mortimer, Oigres D. Bernardinelli, Ann-Christin Pöppler, Steven P. Brown, Eduardo R. deAzevedo, Ray Dupree, Paul Dupree

**Affiliations:** 1 Department of Biochemistry and Leverhulme Centre for Natural Material Innovation, Hopkins Building, Downing Site, University of Cambridge, Cambridge CB2 1QW, UK; 2 Instituto de Física de São Carlos, Departamento de Física e Ciência Interdisciplinar, Universidade de São Paulo, Caixa Postal 369, São Carlos, Sao needs, São Paulo 13660-970, Brazil; 3 Department of Physics, University of Warwick, Coventry CV4 7AL, UK

## Abstract

Exploitation of plant lignocellulosic biomass is hampered by our ignorance of the molecular basis for its properties such as strength and digestibility. Xylan, the most prevalent non-cellulosic polysaccharide, binds to cellulose microfibrils. The nature of this interaction remains unclear, despite its importance. Here we show that the majority of xylan, which forms a threefold helical screw in solution, flattens into a twofold helical screw ribbon to bind intimately to cellulose microfibrils in the cell wall. ^13^C solid-state magic-angle spinning (MAS) nuclear magnetic resonance (NMR) spectroscopy, supported by *in silico* predictions of chemical shifts, shows both two- and threefold screw xylan conformations are present in fresh *Arabidopsis* stems. The twofold screw xylan is spatially close to cellulose, and has similar rigidity to the cellulose microfibrils, but reverts to the threefold screw conformation in the cellulose-deficient *irx3* mutant. The discovery that induced polysaccharide conformation underlies cell wall assembly provides new principles to understand biomass properties.

## Abstract (web summary)

The polysaccharide xylan binds to cellulose microfibrils in the plant cell wall, but the nature of this interaction remains unclear. Here Simmons *et al*. show that while xylan forms a threefold helical screw in solution it forms a twofold screw to bind cellulose microfibrils in the plant cell wall.

Plant secondary cell walls are strong composites of polysaccharides, lignin and proteins that are crucial for plant structure. They provide the most abundant renewable materials on Earth. This lignocellulosic material is therefore the focus of biofuel development^1^, and wood is widely used for many industries such as building construction, paper and packaging. Polysaccharides constitute over 70% of dry cell wall lignocellulosic biomass and can be categorized as cellulose, hemicelluloses and pectins in decreasing order of prevalence (Supplementary Fig. 1)^2^,^3^. Properties such as biomass recalcitrance and timber strength arise from secondary cell wall molecular architecture: the molecular arrangements and conformations of the polysaccharides and other components within the secondary cell wall. Nevertheless, cell wall molecular architecture is very poorly understood.

The semi-crystalline cellulose microfibrils provide the best understood example of molecular architecture in lignocellulose. Its component β-(1→4)-D-glucan chains fold in a twofold helical screw conformation (one 360° twist per 2 glycosidic bonds). The pattern of hydrogen bonding interactions is well understood from X-ray diffraction and magic-angle spinning (MAS) solid-state nuclear magnetic resonance (NMR) studies^4^,^5^,^6^. On the other hand, the intermolecular interactions that occur between cellulose fibrils and hemicelluloses have been a matter of debate for several decades^2^,^7^. Xyloglucan was thought to crosslink microfibrils in primary cell walls. Multidimensional MAS NMR analyses of primary cell walls, pioneered in recent years by Hong and colleagues, have shown that, in contrast, only a minor portion of xyloglucan interacts with cellulose^8^,^9^,^10^,^11^,^12^,^13^,^14^. In secondary cell walls, xylan (Supplementary Fig. 1) and glucomannan are known to bind to cellulose microfibrils, but there is little evidence for the mechanism of this interaction. As the main hemicellulose of eudicot secondary cell walls, the interaction of xylan with cellulose is expected to have the most profound effect on cell wall characteristics and therefore properties such as biomass recalcitrance and timber strength. Indeed, *Arabidopsis* plants with reduced xylan quantity show weakened walls and the plants are unable to develop a vascular system^15^,^16^. Molecular dynamics simulations suggest multiple layers of xylan could envelop microfibrils^17^, and reconstitution studies have revealed that xylan is able to crosslink microfibrils^18^, yet no clear model for the interaction has emerged. On the basis of a conserved pattern of xylan substitution strictly on alternate xylosyl residues, it has recently been proposed that xylan might be able to hydrogen bond to the hydrophilic surfaces of cellulose through folding as a twofold helical screw (Supplementary Fig. 2)^19^,^20^,^21^,^22^. This model is controversial because xylan in solution forms a threefold helical screw^19^,^23^ (one 360° twist per three glycosidic bonds). Despite the conservation of a periodic structure of xylan substitutions throughout seed plants, there is no direct experimental evidence for this model. MAS NMR has the potential to reveal polysaccharide conformations and interactions^11^,^24^,^25^,^26^,^27^. We have therefore recently developed its use to study *Arabidopsis* secondary cell wall^28^, so that mutants can be studied.

By combining *Arabidopsis* molecular genetics and MAS NMR approaches, here we show that xylan interacts intimately with cellulose by adopting a twofold helical screw conformation. The change of xylan fold is induced by, and dependent upon, cellulose fibrils. This provides the first direct experimental evidence for the atomic-scale arrangement of two distinct polymers within plant cell wall biomass, and reveals new principles underlying the remarkable specificity of polysaccharide interactions. Our work provides an understanding of the molecular architectural basis for the biological and mechanical function of cell wall components in plant biomass.

## Results

### Xylan adopts two distinct conformations in the cell wall

**Figure 1:**
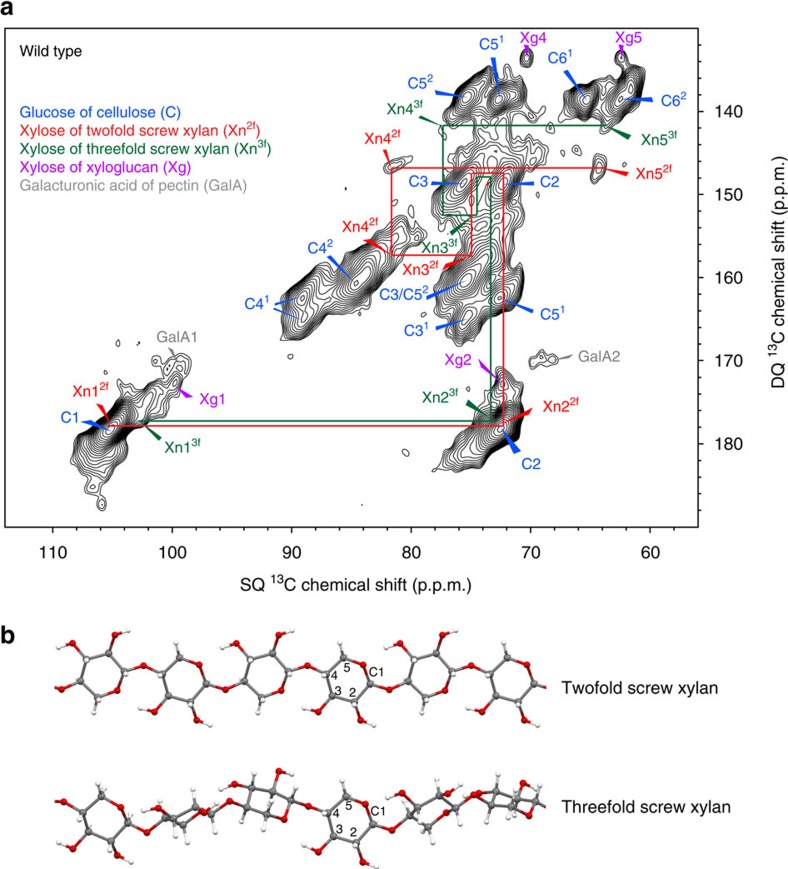
Two distinct xylan populations in twofold screw and threefold screw helical conformations are found in never-dried wild-type *Arabidopsis* stems. (**a**) The carbohydrate region of a refocussed CP-INADEQUATE ^13^C NMR spectrum of never-dried wild-type *Arabidopsis* stems is shown. Red and green labelling illustrates the two distinct xylan conformations, shown clearly here by the distinct xylan carbon 4 (Xn4) and Xn5 shifts. Xn1, Xn2 and Xn3 also differ between conformations but not all differences are readily distinguished in this CP spectrum (see Fig. 4 and Table 1 for full assignments). The spectrum was recorded at a ^13^C Larmor frequency of 125.8 MHz and a MAS frequency of 10 kHz using a total spin-echo duration of 1.2 ms. (**b**) *In silico* DFT-optimized xylan structures from which ^13^C chemical shifts in Table 1 were calculated.

To analyse the structure of xylan in never-dried cell walls by ^13^C MAS NMR, a chamber to grow uniformly labelled *Arabidopsis* plants in ^13^CO_2_ was designed and constructed (Supplementary Fig. 3). Fresh and unprocessed stems were directly analysed, ensuring that the native arrangement of the cell wall polysaccharides was preserved. Figure 1a shows a two-dimensional (2D) refocused INADEQUATE^25^,^29^,^30^ MAS NMR spectrum of wild-type *Arabidopsis* stems using cross polarization (CP) excitation, which emphasizes the more rigid components. In this experiment directly bonded nuclei appear at the same double-quantum (DQ) chemical shift that corresponds to the sum of the two single-quantum (SQ) chemical shifts. The spectrum shows broad line widths of ∼2 p.p.m. indicating the relative rigidity of the polysaccharides and a range of different environments. As expected, the major peaks in the spectrum are from glucose in the two main cellulose environments (C^1^: often assigned to crystalline and/or internal glucan chains; and C^2^: often assigned to amorphous and/or surface glucan chains)^8^,^9^,^10^,^11^,^12^,^28^. Surprisingly, two distinct xylan carbon 4 (Xn4) peaks are clearly visible in the spectrum (82.2 and 77.4 p.p.m.), each coupled to Xn5 (64.3 and 63.9 p.p.m.). The existence of the two discrete Xn4 shifts indicates the presence of two distinct conformations of xylan in the fresh hydrated cell walls. This is in contrast to the range of shifts observed for Xn4 in dried *Arabidopsis* stems^28^, suggesting that drying had a substantial effect on native xylan conformations.

Xylan in solution forms a threefold helical screw^19^,^23^. The 77.4 p.p.m. Xn4 and 63.9 p.p.m. Xn5 shifts are very similar to the solution state xylan Xn4 shift (Table 1), suggesting that they arise from threefold screw xylan in the cell wall, so we named them Xn4^3f^ and Xn5^3f^, respectively. It has been proposed that some xylan may fold as twofold screw in the cell wall^19^,^20^,^21^,^22^. A change in torsion angle around the glycosidic bond (which yields different helical conformations) can alter the chemical shifts of polysaccharides, and in (1→4)-linked glycans changes in torsion angle has been shown to affect carbon 1 and carbon 4 resonances particularly^27^. The 82.2 p.p.m. Xn4 shift seen in the fresh cell walls might therefore arise from a change in conformation of the xylan to a twofold screw. To estimate the shift differences between the twofold screw and threefold screw xylan conformations, we performed *in silico*^13^C chemical shift prediction using gauge including projector augmented waves density functional theory (DFT)^31^,^32^ (Fig. 1b). As expected, the two carbons involved in the glycosidic bond–Xn1 and Xn4–were predicted to alter the most after changing conformation (Table 1 and Supplementary Tables 1 and 2). Xn1 and Xn4 are predicted to be substantially shifted to higher p.p.m. in a twofold screw compared with a threefold screw conformation. Dominant cellulose signals mask the twofold Xn1 signal (Xn1^2f^) in the CP-refocused INADEQUATE spectrum (Fig. 1; Xn1 assigned below). However, the presence of the Xn4 signal at the higher (82.2 p.p.m., Xn4^2f^) ^13^C chemical shift, covalently linked to an Xn5 signal (Xn5^2f^), indicates that xylan forms a twofold screw in the cell wall.

### Cellulose is required for xylan twofold screw conformation

**Figure 2:**
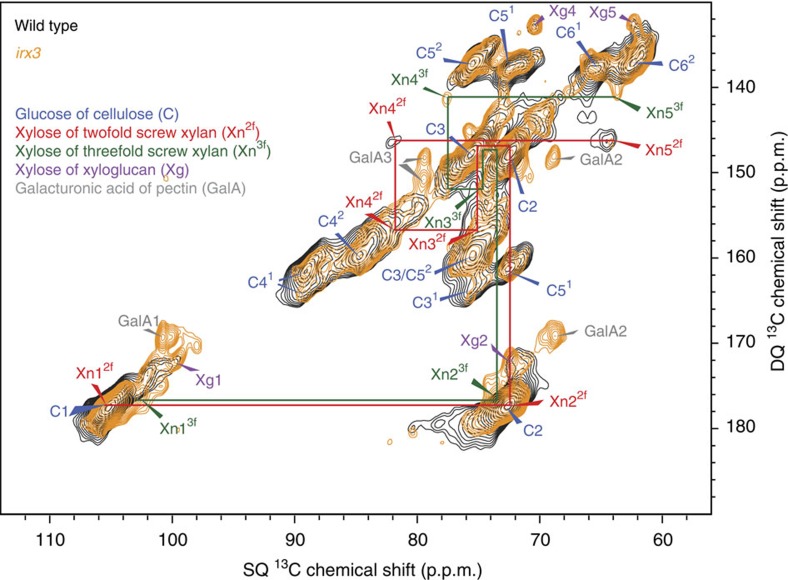
Twofold screw xylan predominates in wild type but is barely detectable in the cellulose-deficient mutant *irx3.* An overlay is shown of carbohydrate regions of refocussed CP-INADEQUATE ^13^C NMR spectra of wild-type and *irx3*. Red and green labelling illustrates the two distinct xylan conformations shown clearly here by the distinct Xn4 and Xn5 shifts. The spectra were recorded as in Fig. 1, using a total spin-echo duration of 2.2 ms.

**Figure 3:**
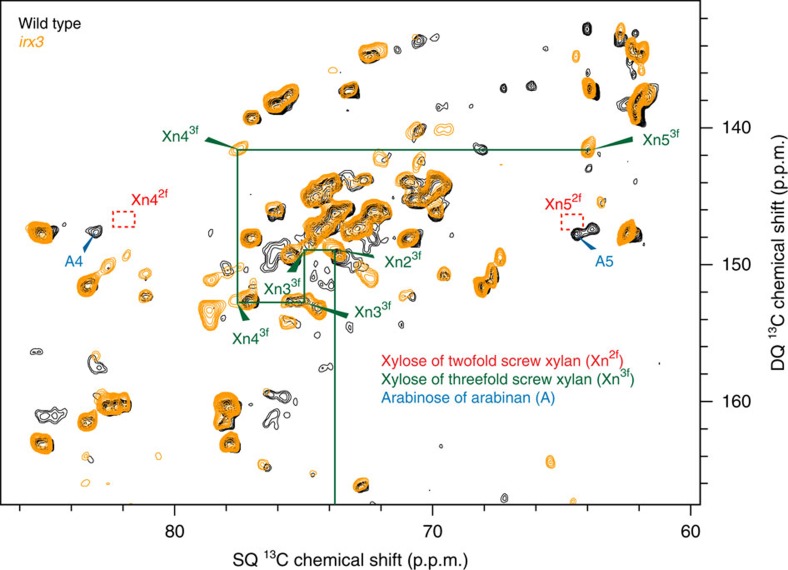
Threefold screw xylan is abundant in *irx3* but not in wild type as shown by analysis of mobile cell wall components. An overlay is shown of ^13^C refocussed DP-INADEQUATE ^13^C NMR spectra of wild type and *irx3* never-dried *Arabidopsis* stems using a 1.9 s recycle time, which emphasize relatively mobile species. The carbohydrate region of the spectrum is expanded to focus on the Xn4→Xn5 connection. This shows that threefold xylan is abundant in *irx3,* and the twofold xylan is relatively immobile and so is not detected in wild type or *irx3* mutant cell walls. Xylose of threefold xylan is highlighted in green. Dotted red squares indicate the absence of the twofold screw Xn4→Xn5 pair. The change in some arabinose and pectin structures in the plants may be due to altered growth of the dwarfed *irx3* plant. The spectrum of the full carbohydrate region is shown in Supplementary Figure 5, and was recorded with a spin-echo duration of 2.2 ms at a ^13^C Larmor frequency of 213.8 MHz and a MAS frequency of 12 kHz.

Xylan might be induced to adopt the twofold screw conformation upon binding to cellulose in the cell wall^19^,^20^,^21^,^22^. Indeed, 82–84 p.p.m. peaks have previously been proposed to arise from cellulose-aggregated xylan^33^,^34^,^35^,^36^. Therefore, we next investigated whether the twofold screw xylan is dependent on cellulose in the cell wall. The *irx3 Arabidopsis* mutant is deficient in secondary cell wall cellulose synthesis and is consequently dwarfed, but the plant continues to synthesize other cell wall components including xylan^37^,^38^. Because it has much reduced secondary cell wall cellulose, it provides a useful tool to study the importance of cellulose for xylan conformation. One-dimensional MAS NMR of *irx3* stems confirmed depletion in this mutant of cellulose and the relative increase in other cell wall polysaccharides, including xyloglucan (Xg) and pectin (GalA) (Supplementary Fig. 4). Interestingly, a CP-refocused INADEQUATE spectrum showed a profound difference in the xylan conformation in *irx3* compared with wild type. While twofold screw xylan (Xn4^2f^ peak) was by far the more prevalent of the two conformations in wild type (Figs 1 and 2), in *irx3* it was barely detectable (Fig. 2). In contrast, almost all *irx3* xylan was found in the threefold conformation. To study the more mobile cell wall components in *irx3* and wild-type stems, direct polarization (DP)-INADEQUATE spectra with a short recycle delay were acquired. The abundance of the Xn4^3f^ peak showed that relatively mobile threefold screw xylan is far more prevalent in *irx3* than in the wild type (Fig. 3 and Supplementary Fig. 4). Indeed, this mobile threefold screw xylan was not detected in wild-type plants. Neither the twofold screw xylan nor cellulose was seen in the DP-INADEQUATE spectra of wild-type plants, indicating that the twofold screw xylan, like cellulose, is relatively immobile. The absence of the twofold screw xylan in *irx3* shows that the change in xylan conformation from threefold to twofold screw is cellulose-dependent.

**Figure 4:**
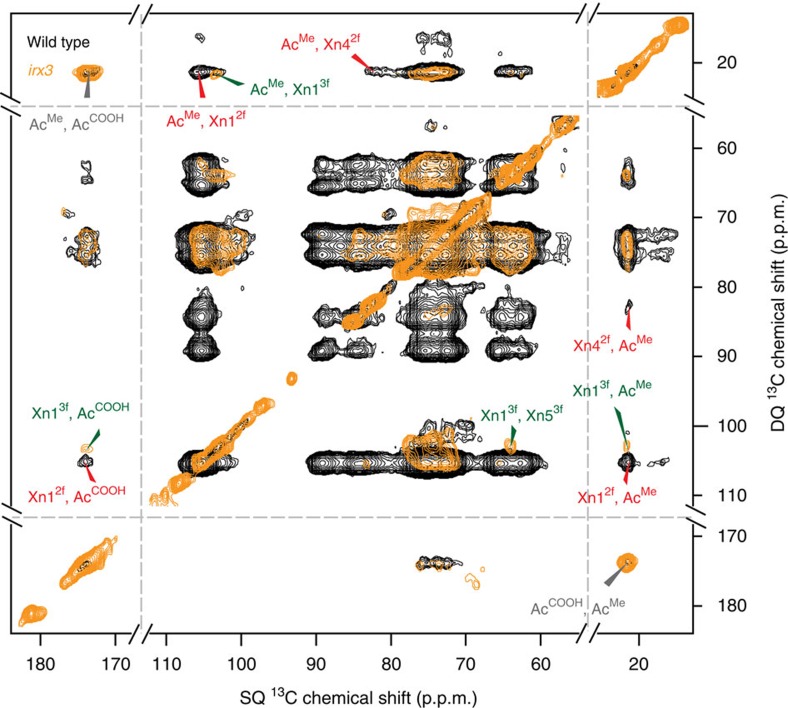
Twofold and threefold screw xylan have distinct carbon 1 shifts. An overlay is shown of ^13^C PDSD NMR spectra of wild type (CP-PDSD 1 s mixing time) and *irx3* (DP-PDSD, 1.5 s mixing time) never-dried *Arabidopsis* stems. Wild-type and *irx3* show two distinct xylan conformations: in wild-type plants Xn1^2f^ resonates at 105.2 p.p.m., whereas in *irx3* Xn1^3f^ is at 102.6 p.p.m. These long mixing time spectra were recorded at a ^13^C Larmor frequency of 213.8 MHz and a MAS frequency of 12 kHz.

The presence of abundant threefold screw xylan in *irx3* cell walls gave us the opportunity to investigate further the Xn1^2f^ and Xn1^3f 13^C chemical shifts, given the DFT prediction of substantial change in Xn1 (Table 1). Using the knowledge that xylan is highly acetylated, the shifts of carbon atoms close to acetate moieties in the cell wall were investigated using proton-driven spin diffusion (PDSD) experiments, which measure through-space proximities of different moieties^39^, with longer mixing times probing longer distances. Figure 4 shows a comparison of long mixing time PDSD spectra of wild-type plants (a CP spectrum where mainly twofold xylan is visible), with *irx3* (a DP-PDSD spectrum where only threefold xylan is visible). Acetate methyl groups (21.6 p.p.m.) are spatially close to acetate carbonyl groups (173.6 p.p.m.) and also to Xn1–Xn5. Interestingly, the Xn1^3f^ shift in *irx*3 is 102.6 p.p.m. as seen in DP INADEQUATE and in solution xylan, but in wild-type Xn1^2f^ is significantly shifted to the higher ^13^C chemical shift of 105.2 p.p.m.. Because a clear increase in Xn1^2f^ shift from threefold to twofold was predicted by the calculations (Table 1), this observation further supports the assignment of the conformation which predominates in wild type as twofold screw, and the conformation which predominates in *irx3* as threefold. Notably, the Xn1^2f^ shift of 105.2 p.p.m. is very similar to cellulose C1 at 105.1 p.p.m., which also exists in a twofold screw.

### Xylan with a twofold screw conformation is bound to cellulose

**Figure 5:**
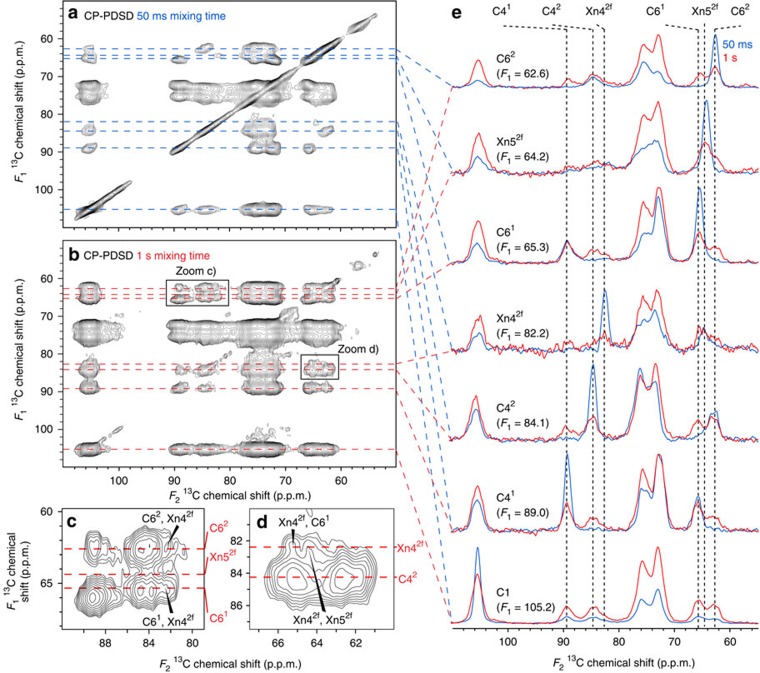
Twofold screw xylan is found in close proximity to cellulose in never-dried wild-type *Arabidopsis* stems. Carbohydrate regions are shown of *z*-filtered CP-PDSD^39^ ^13^C NMR spectra of never-dried wild-type *Arabidopsis* stems using a total *z* period of 1.005 s. (**a**) 50 ms mixing time spectrum. (**b**) 1 s mixing time spectrum. (**c**) Zoom of 1 s mixing time spectrum showing cellulose→xylan connections. (**d**) Zoom of 1 s mixing time spectrum showing xylan→cellulose connections. (**e**) One-dimensional slices from 50 ms (blue) and 1 s (red) mixing time spectra. The spectra were recorded as in Fig. 4.

To determine whether the twofold screw xylan is cellulose-bound, spatial proximities of molecules in the cell wall were investigated using a relaxation compensated *z*-filtered version of the CP-PDSD experiment^40^ (Fig. 5). In the experiment with a short 50 ms mixing time, cross peaks indicative of short-distance intramolecular spatial relationships were observed, for example, within cellulose domain 1 (C4^1^-C6^1^; 89.0 p.p.m.-65.3 p.p.m.), cellulose domain 2 (C4^2^-C6^2^; 84.1 p.p.m. to 62.6 p.p.m.) and twofold screw xylan (Xn4^2f^-Xn5^2f^; 82.2 p.p.m. to 64.2 p.p.m.; Supplementary Table 1). In the experiment with a longer, 1 s, mixing time cross peaks indicative of both short and longer distance intra- and intermolecular spatial relationships were observed. Interestingly, cross peaks were seen between xylan and cellulose, indicating their close spatial proximity. However, xylan cross peaks to cellulose domain 1 (for example, Xn4^2f^→C6^1^; Fig. 5c,d), which is often assigned to internal cellulose glucan chains, were greater than those to cellulose domain 2 (for example, Xn4^2f^→C6^2^ Fig. 5c), which is often assigned to surface cellulose glucans. This suggests that xylan coating of cellulose surface chains causes a change in glucan NMR shift, perhaps because these glucans adopt an interior-like conformation, akin to a recent proposal for cellulose: hemicellulose interactions in primary cell walls^13^. The similar signal strengths of the intermolecular Xn4^2f^→C6^1^ and the intramolecular Xn4^2f^→Xn5^2f^ peaks indicate that almost all of the twofold screw xylan is spatially close to cellulose. In contrast at this mixing time the cross peaks showing spatial proximities between cellulose domains (for example, C4^1^→C6^2^ and C6^1^→C6^2^) are not yet as strong as those showing spatial proximities within cellulose domains (for example, C4^1^→C6^1^). Although spin diffusion in a fully ^13^C labelled system is complex, the presence of the relatively large Xn4^2f^→C6^1^ cross peak means that we can place an upper limit on the distance between the twofold screw xylan and cellulose: twofold screw xylan can be no farther than the width of a microfibril from the cellulose surface. Hence, the PDSD experiments indicate that the twofold screw xylan is bound to cellulose.

**Figure 6:**
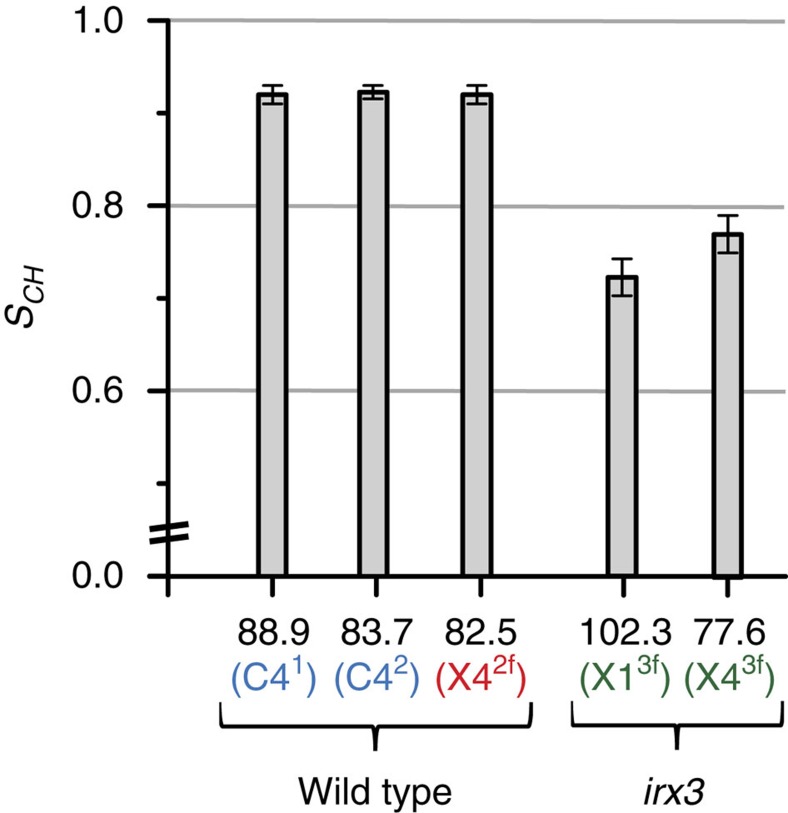
Cellulose and twofold screw xylan are similarly rigid, while threefold screw xylan is more mobile. CP-DIPSHIFT MAS NMR analysis of the order parameter, *S*_CH_, in wild-type and *irx3* stems is shown; this reports on the molecular mobilities of cell wall moieties. The labelling refers to the presumed major contributing species. Refer to Supplementary Fig. 4 for chemical shift assignments of the One-dimensional spectra. See Supplementary Fig. 6 for data fits. The error bars were determined by simulation of the DIPSHIFT curves shown in Supplementary Fig. 6b with varying parameters. The error bar represents the maximum change in parameter before there is a clear deviation of simulated from experimental values. The order parameters for all major peaks are shown in Supplementary Fig. 7. Experiments were carried out at a ^13^C Larmor frequency of 125.8 MHz and a MAS frequency of 7.813 kHz.

As a further means of probing xylan: cellulose interactions we analysed the molecular mobility of these polysaccharides by measuring the ^13^C *T*_1_ relaxation time (Supplementary Table 3), which is sensitive to motion on the nanosecond timescale, and the dipolar order parameter *S*_*CH*_ (0≤*S*_*CH*_≤1) which is sensitive to motion on the microsecond timescale. Both DP and CP DIPSHIFT^41^ experiments were used to measure the order parameter as there is more than one contribution to the signal for many chemical shifts. The DP DIPSHIFT with 20 s recycle delay was quantitative with all of the species contributing, whereas the CP DIPSHIFT preferentially measured the immobile components such as cellulose and any cellulose-bound xylan. Figure 6 shows a comparison of the CP order parameters representative of cellulose, twofold xylan and threefold xylan, all obtained from the dephasing curves shown in Supplementary Fig. 6, for both wild-type and *irx3* samples. Cellulose-bound xylan would be expected to exhibit less motion (demonstrated by a larger order parameter) than unbound xylan. The order parameter of twofold xylan (specifically measuring Xn4^2f^ in wild-type stems) is comparable to that of both cellulose domains (specifically measuring C4^1^ and C4^2^ in wild-type stems), indicating relatively little motion. In contrast, for threefold xylan (specifically measuring Xn1^3f^ and Xn4^3f^ in *irx3* stems) the order parameter is significantly reduced, indicating more motion. Consistent with this, the *T*_1_ of Xn4^2f^ (82.5 p.p.m.) in wild type is very similar to cellulose values whereas Xn1^3f^ in *irx3* has shorter *T*_1_ (see Supplementary Table 3). Therefore, twofold screw xylan is far less mobile than threefold screw xylan, and has a similar rigidity to cellulose.

The CP-INADEQUATE and DP-INADEQUATE experiments together indicated that the large majority of xylan in the secondary cell walls of wild-type plants folds as twofold screw, with only a minor proportion of xylan detected in a relatively immobile threefold screw conformation. The PDSD and DIPSHIFT experiments, together with the requirement for cellulose revealed by *irx3*, indicated that essentially all the twofold screw xylan is bound to cellulose. These results are consistent with the finding that the majority of xylan has substitution patterning that is compatible with binding to the hydrophilic surfaces of cellulose^19^,^20^,^21^. However, we do not yet know the relative amounts of the xylan bound to the hydrophilic vs hydrophobic surfaces of the fibrils. Moreover, the proportion of hydrophilic and hydrophobic surfaces on the cellulose microfibril structure is not yet known. In the secondary cell walls of eudicots, the ratio between quantity of xylan and cellulose is approximately 2:3 (ref. 42). Our results therefore suggest that there is likely sufficient xylan to coat the hydrophilic surfaces of cellulose microfibrils. Nevertheless, further work is necessary to determine the extent of xylan binding to the different fibril surfaces.

## Discussion

The molecular arrangement of plant cell wall components has been an unresolved question for decades. We have now shown that xylan is induced to assemble on cellulose fibrils as a twofold helical screw in secondary cell walls. This is the first direct evidence for the atomic-scale model of the interactions between cellulose and xylan in plant cell walls (Supplementary Fig. 2)^19^,^20^. In this model, xylan hydrogen bonds with cellulose microfibril hydrophilic surfaces by forming a flattened twofold helical screw. This effectively extends the semi-crystalline cellulose microfibril size and alters its properties. Coating of the hydrophilic faces of cellulose microfibrils with the acetylated and glucuronosylated form of xylan found in dicot cell walls may lead the otherwise hydrophilic surfaces to be relatively hydrophobic (because of acetate groups) and acidic (because of glucuronic acid groups). This may be of profound importance to cell wall architecture, for example influencing interactions between the cellulose-xylan fibrils, and altering binding to the other components of the secondary cell wall, especially lignin. Xylan folded onto cellulose surfaces is likely to be resistant to the action of microbial hydrolases which cleave threefold screw xylan. It remains unknown whether some xylan may also bind to the hydrophobic surface of fibrils. The evenly spaced glucuronosyl and acetyl substitutions of xylan^19^,^20^,^21^,^22^ are important for allowing xylan to bind to the hydrophilic surfaces of cellulose. Because xylan from gymnosperms to eudicots has retained this even pattern of substitution^22^, we believe the assembly of xylan onto cellulose revealed in this work is likely to be conserved across land plants. Through discovery of the specific interaction of these two polysaccharides, this work therefore reveals a fundamental principle in assembly of plant cell walls which likely extends to other polysaccharide interactions and the extracellular matrix of many organisms.

A description of the nature of the interactions between cell wall components is crucial for many uses of plants as a renewable resource. It helps us understand the basis of plant recalcitrance to digestibility and deconstruction, which is of importance in dietary fibre, animal fodder and in biofuel production. These findings will also be important in improving processes using materials made from plants, such as the paper industry and building construction. Our studies of cell wall molecular architecture therefore provide a new twist for exploitation of plant materials.

## Methods

### Growth chamber and plant preparation

*irx3-7* seeds were kindly donated by Simon Turner (University of Manchester). Seeds were surface sterilized and sown on 0.8% (w/v) agar, 0.5 9 Murashige and Skoog salts including vitamins (Sigma, http://www.sigma.com) and sucrose (1% w/v). Following stratification for 48 h at 4 °C in the dark, plates were transferred to a growth room (20 °C, 100 μmol m^−2^ s^−1^, 24 h light, 60% humidity). Optimaxx fibre rockwool (Cultilene, Netherlands) was cut into slabs and laid ∼8 cm deep in a growth tray. Hydroponics solution (2 mM MgSO_4_, 2 mM CaNO_3_, 50 μM FeEDTA, 5 mM KNO_3_, 2.5 mM K_2_HPO_4_/KH_2_PO_4_ pH 5.5, 70 μM H_3_BO_4_, 14 μM MnCl_2_, 0.5 μM CuSO_4_, 1 μM ZnSO_4_, 0.2 μM NaMoO_4_, 10 μM NaCl and 0.1 μM CoCl_2_) was poured into the tray until half-way up. The rockwool was then covered with foil. Holes were pierced into the foil and rockwool and seedling were placed in, ensuring that roots made direct contact with rockwool. A growth chamber was constructed following Chen *et al*.^43^ illustrated in Supplementary Fig. 3. Compressed air was scrubbed of CO_2_ using calcium oxide before ^13^CO_2_ was mixed back in at a concentration of 500 p.p.m. before entering the growth chamber. Plants were grown in a 50% humidity and 24 °C environment for 6–8 weeks. ^13^C enrichment was measured by analysing xyloglucan oligosaccharides yielded from xyloglucan endoglucanase digestion of stems using matrix-assisted laser desorption/ionization–time of flight mass spectrometry. Typical enrichments were 90–95%. Roughly 35 mg of the bottom third parts of five to ten stems were chopped and packed into a 3.2 mm Magic-Angle Spinning NMR rotor. For wild-type, five biological replicates (of at least five plants) were grown, each of which was analysed at least once; for *irx3*, two biological replicates (of at least five plants) were grown, each of which was analysed at least once. On the basis of the relative content of cellulose, pectin, xyloglucan and xylan observed in the wild-type and *irx3* samples by NMR, we estimate the stem material contained a substantial but minority proportion of primary cell walls.

### NMR chemical shift predictions

The NMR shift calculations were carried out using the CASTEP code^44^ with the PBE^45^ exchange correlation functional. The 10 molecule (two- and threefold) MD xylan structures generated earlier^19^ were first geometry optimized using a cutoff energy of 800 eV with a *k* point grid of 2 × 2 × 1 and a fixed unit cell of 10 × 10 × 60 Å. The NMR shielding was calculated for the resulting structures using the gauge including projector augmented waves^31^,^32^ method. The reference used to convert shielding to shift, 167.7 p.p.m., was determined by assuming that the threefold structure is very similar to xylan in solution (see Supplementary Table 2). Its value does not affect the shift differences given in Table 1.

### Solid-state NMR

Solid-state MAS NMR experiments were performed on Bruker (Karlsruhe, Germany) 850 and 500 MHz Advance III solid-state NMR spectrometers, operating at ^1^H and ^13^C Larmor frequencies of 850.2 and 213.8 MHz and 500.1 and 125.8 MHz, respectively, using 3.2 mm double-resonance MAS probes. Experiments were conducted at room temperature at MAS frequencies of 12–14 kHz at the higher field and 10 kHz on the 500 MHz spectrometer unless otherwise stated. The ^13^C chemical shift was determined using the carbonyl peak at 177.8 p.p.m. of L-alanine as an external reference with respect to tetramethylsilane (TMS); 90° pulse lengths were typically 3.5 μs (^1^H) and 4.2 μs (^13^C). Both ^1^H−^13^C cross-polarization (CP) with ramped (70–100%) ^1^H rf amplitude^46^ and a contact time of 1 ms and direct polarization (DP) were used to obtain the initial transverse magnetization. While CP emphasizes the more rigid material, a short, 1.9 s, recycle delay DP experiment was used to preferentially detect the mobile components and a 20 s delay was used for quantitative experiments. SPINAL-64 decoupling^47^ was applied during acquisition at a ^1^H nutation frequency of 70–80 kHz. Two-dimensional double-quantum (DQ) correlation spectra were recorded using the refocused INADEQUATE pulse sequence which relies upon the use of isotropic, scalar J coupling to obtain through-bond information regarding directly coupled nuclei^25^,^29^,^30^. The carbon 90° and 180° pulse lengths were 4 and 8 μs, respectively with 2*τ* spin-echo evolution times for a (*τ–π–τ*) spin-echo of 1.2 to 4.4 ms and the SPINAL-64 ^1^H decoupling^47^ was applied during both the evolution and signal acquisition periods. The acquisition time in the indirect dimension (*t*_1_) was 4.5 -5.0 ms with a recycle delay of 1.5 and 1.9 s for CP and DP experiments, respectively. Intermolecular contacts were probed using 2D ^13^C−^13^C ^1^H driven spin diffusion (PDSD) experiments^39^,^40^ with mixing times of 50 ms to 1.5 s. For both refocused INADEQUATE and PDSD experiments, the spectra were obtained by Fourier transformation into 2 K (F2) × 1 K (*F*_1_) points with exponential line broadening of 80 Hz (CP) and 40 Hz (DP) in *F*_2_ and squared sine bell processing in *F*_1_. The ^13^C spin lattice relaxation time, *T*_1_, was measured at 125.8 MHz using saturation recovery following a comb of 30 pulses and echo acquisition. The dipolar order parameter, *S*_*CH*_, was determined using the 2D ^13^C−^1^H dipolar chemical shift (DIPSHIFT) experiment^41^ whilst spinning at 7.813 kHz on the 500 MHz spectrometer. The ^1^H homonuclear coupling was suppressed using the frequency switched Lee-Goldberg sequence^48^ with a ^1^H nutation frequency of 80 kHz. Measurements were taken with CP and with DP excitation with recycle delays of 2 and 20 s, respectively. All spectra obtained were processed and analysed using Bruker Topspin version 3.2.

### DIPSHIFT calculations

DIPSHIFT experiments were simulated using a home written program in Origin.c. The DIPSHIFT curves were calculated as a simple evolution of the ^13^C magnetization under C−H dipolar coupling and MAS. The effective C−H coupling for CH_2_ units was obtained using the expressions reported by Terao *et al*.^49^ This provides a reliable measurement of the effective C−H coupling in the fast and rigid motion limits, as confirmed by comparison with full spin dynamics simulations^50^.

### Data availability

The solid-state NMR data and scripts for DIPSHIFT simulations are available at http://dx.doi.org/10.17863/CAM.5896. The authors declare that all other relevant data supporting the findings of this study are available within the article and its Supplementary Information files or on request from the corresponding authors.

## Additional information

**How to cite this article:** Simmons, T. J. *et al*. Folding of xylan onto cellulose fibrils in plant cell walls revealed by solid-state NMR. *Nat. Commun.* **7,** 13902 doi: 10.1038/ncomms13902 (2016).

**Publisher's note:** Springer Nature remains neutral with regard to jurisdictional claims in published maps and institutional affiliations.

## Supplementary Material

Supplementary Information Supplementary Figures, Supplementary Tables and Supplementary References.

Supplementary Information Peer Review File

## Figures and Tables

**Table 1:** Comparison of observed and *in silico* predicted xylan ^13^C chemical shifts.

	**Experimental** ^**13**^**C chemical shifts (p.p.m.)**			^**13**^**C chemical shift difference (p.p.m.)**	
	**In cell wall**		In solution*	**Observed in cell wall**	**Predicted from** ***in silico***† **modelling**
	**Twofold**‡	**Threefold**§	**Threefold**	**Twofold minus Threefold**	
Xn1	105.2	102.6	102.5	+2.6	+4.0
Xn2	72.3	73.7	73.5	−1.4	−2.9
Xn3	(75.2)	74.7	74.4	(+0.5)	+1.3
Xn4	82.2	77.4	77.2	+4.8	+8.2
Xn5	64.3	63.9	63.7	+0.4	+1.2

DFT, density functional theory; MAS, magic-angle spinning; NMR, nuclear magnetic resonance; PDSD, proton-driven spin diffusion.

^*^Threefold screw xylan solution NMR ^13^C chemical shifts are averaged data for non-acetylated residues^19^. The MAS NMR ^13^C chemical shift for twofold screw Xn3 is less certain as it is very similar to that of cellulose C3.

^†^The *in silico* prediction of ^13^C chemical shift differences used 10 residue two- and threefold DFT-optimized molecular dynamics-generated xylan structures.

^‡^Twofold screw xylan MAS NMR ^13^C chemical shifts taken from wild-type CP-refocused INADEQUATE and PDSD spectra.

^§^Threefold screw cell wall xylan MAS NMR ^13^C chemical shifts taken from *irx3* DP-refocused INADEQUATE spectra.
