# Supplementary material for: Folding of xylan onto cellulose fibrils in plant cell walls revealed by solid-state NMR
Source: Nat Commun. 2016 Dec 21;7:13902. doi: 10.1038/ncomms13902 (PMC5187587; doi:10.1038/ncomms13902)
Supplement: Supplementary Information — Supplementary Figures, Supplementary Tables and Supplementary References. [file ncomms13902-s1.pdf]

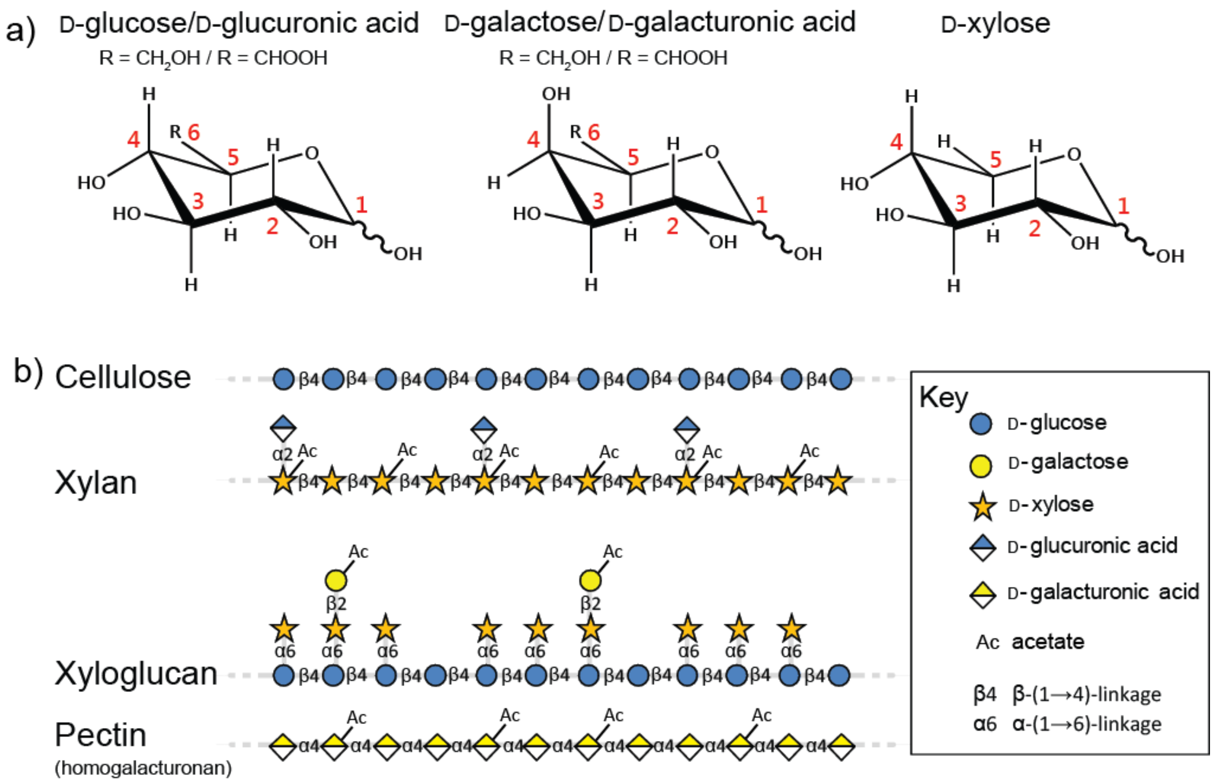

Supplementary Figure 1: Carbohydrate structures described in the manuscript. a, atomic detail structure of the key monosaccharides described in this paper, showing the structural similarity between different sugars e.g. glucose and xylose. Glucuronic acid is further regularly found 4-O-methyl-etherified, and galacturonic acid found C-6 methyl-esterified. **b**, molecular detail structure of the key polysaccharides described in this paper.

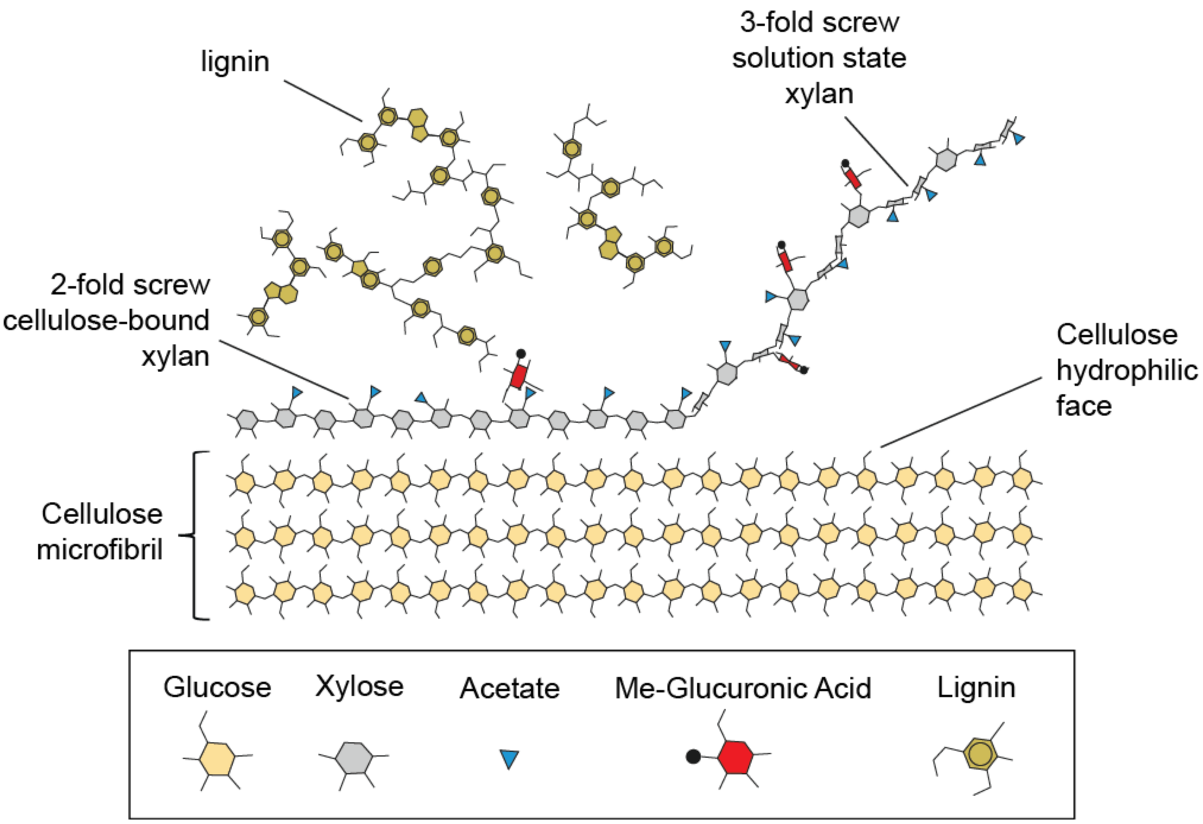

Supplementary Figure 2: Cellulose : xylan interaction model. Model of cellulose : xylan interactions in which non-cellulose-bound xylan exists in its characteristic 3-fold helical screw, while binding of xylan to the cellulose surface induces it to change conformation into a 2-fold helical screw. On the majority of the length of the xylan chain glucuronosyl and acetyl modifications can only be found on every other backbone residue. Because of this, when induced to fold into a 2-fold helical screw, they all point away from the surface of the cellulose microfibril, displaying a relatively hydrophobic (owing to acetate groups) and acidic (owing to glucuronic acid groups) surface. This could thereby mediate interactions between the hydrophilic surfaces of cellulose and hydrophobic cell wall components such as lignin.

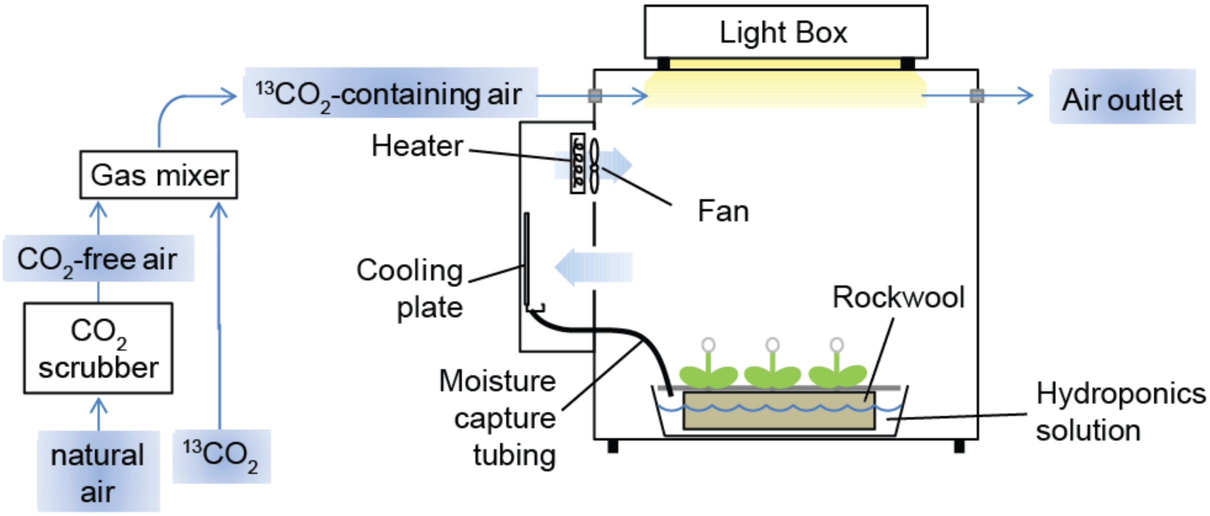

Supplementary Figure 3: Model of the growth chamber. The growth chamber was constructed based on the description by Chen et al.[1] Compressed air is scrubbed of CO_2 using calcium oxide before CO_2-free air is mixed with $^{13}CO_2$ to 500 ppm and is directed into the chamber. The chamber is a sealed environment but for the inlet and a single outlet. Inside the chamber, plants are grown on a bed of rockwool soaked in hydroponics solution. Inside the chamber humidity is controlled by a fan blowing air onto a cold plate; condensed water is directed back into the growth tray via tubing. Temperature is regulated using a heater.

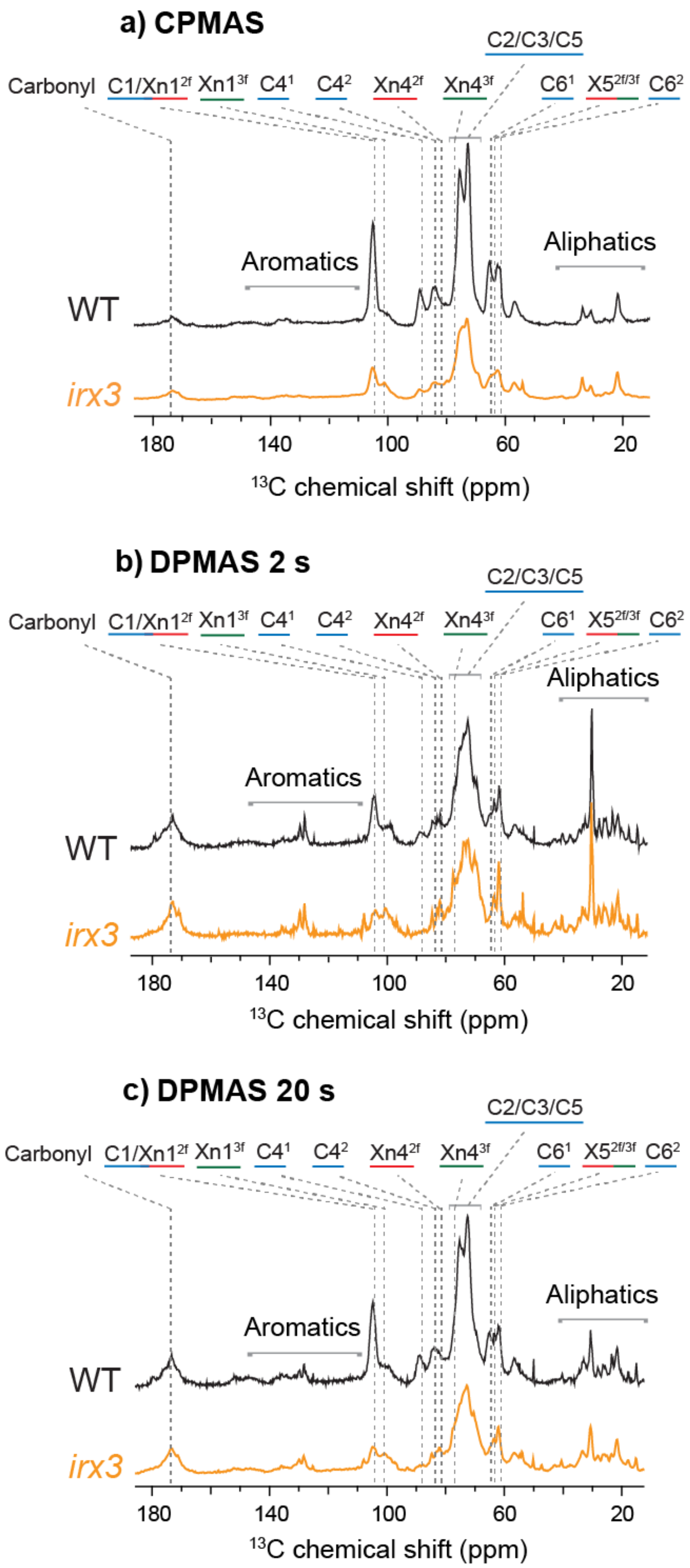

Supplementary Figure 4: 1D ^{13}C MAS NMR spectra from wild type and *irx3*. a, CP MAS NMR spectra showing preferentially the immobile species. **b**, DP MAS NMR spectra with 2 s recycle delay showing preferentially the mobile species. **c**, DP MAS NMR spectra with 20 s recycle delay showing quantitative signal intensities. All spectra taken on a 500 MHz spectrometer. Experiments were carried out at a ^{13}C Larmor frequency of 125.8 MHz and a MAS frequency of 10 kHz.

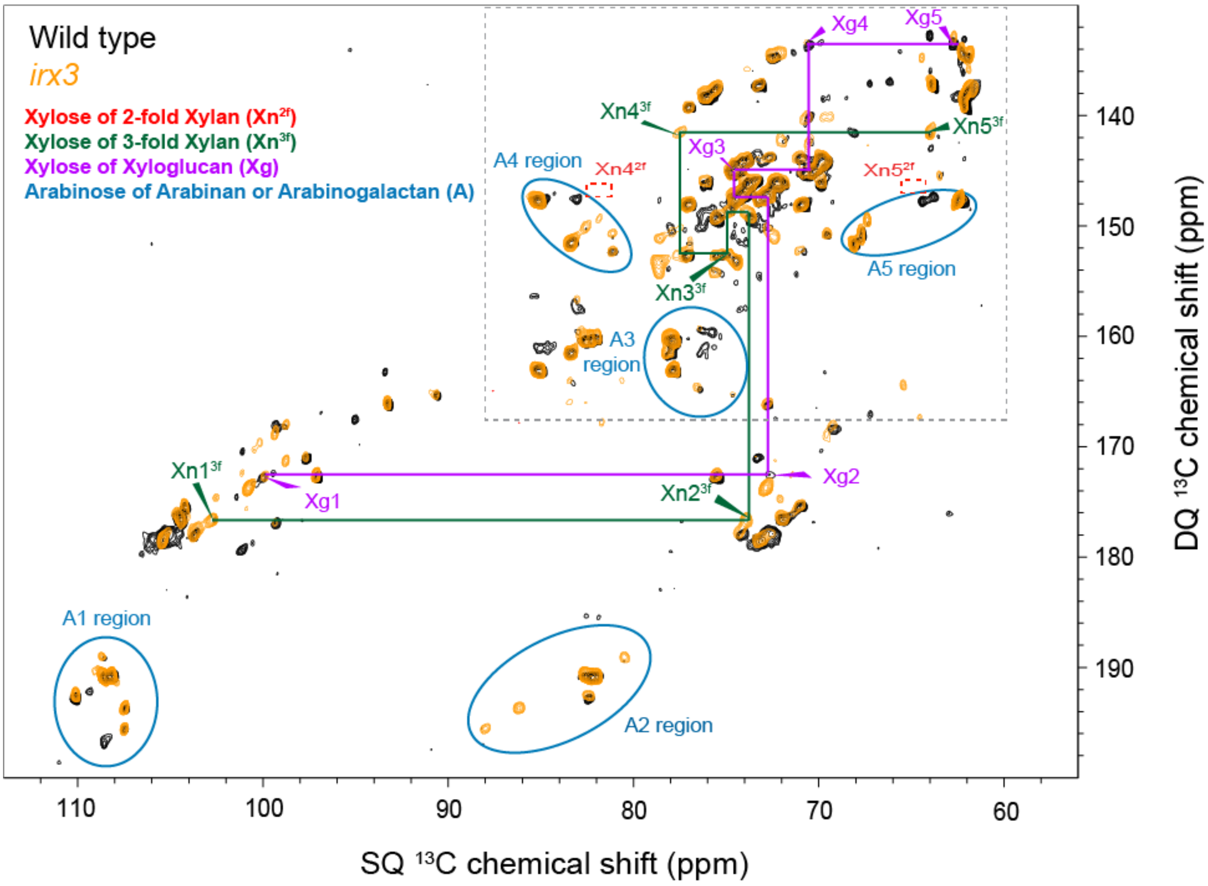

Supplementary Figure 5: Full carbohydrate region of refocussed DP-INADEQUATE spectra of wild type and *irx3*. The area inside the dashed box is shown in Fig. 3. Spectra taken with a recycle delay of 1.9 s to emphasise the mobile components. The arabinose regions are those identified by Wang et al.[2] Experiments were carried out at a ^{13}C Larmor frequency of 213.8 MHz and a MAS frequency of 12 kHz.

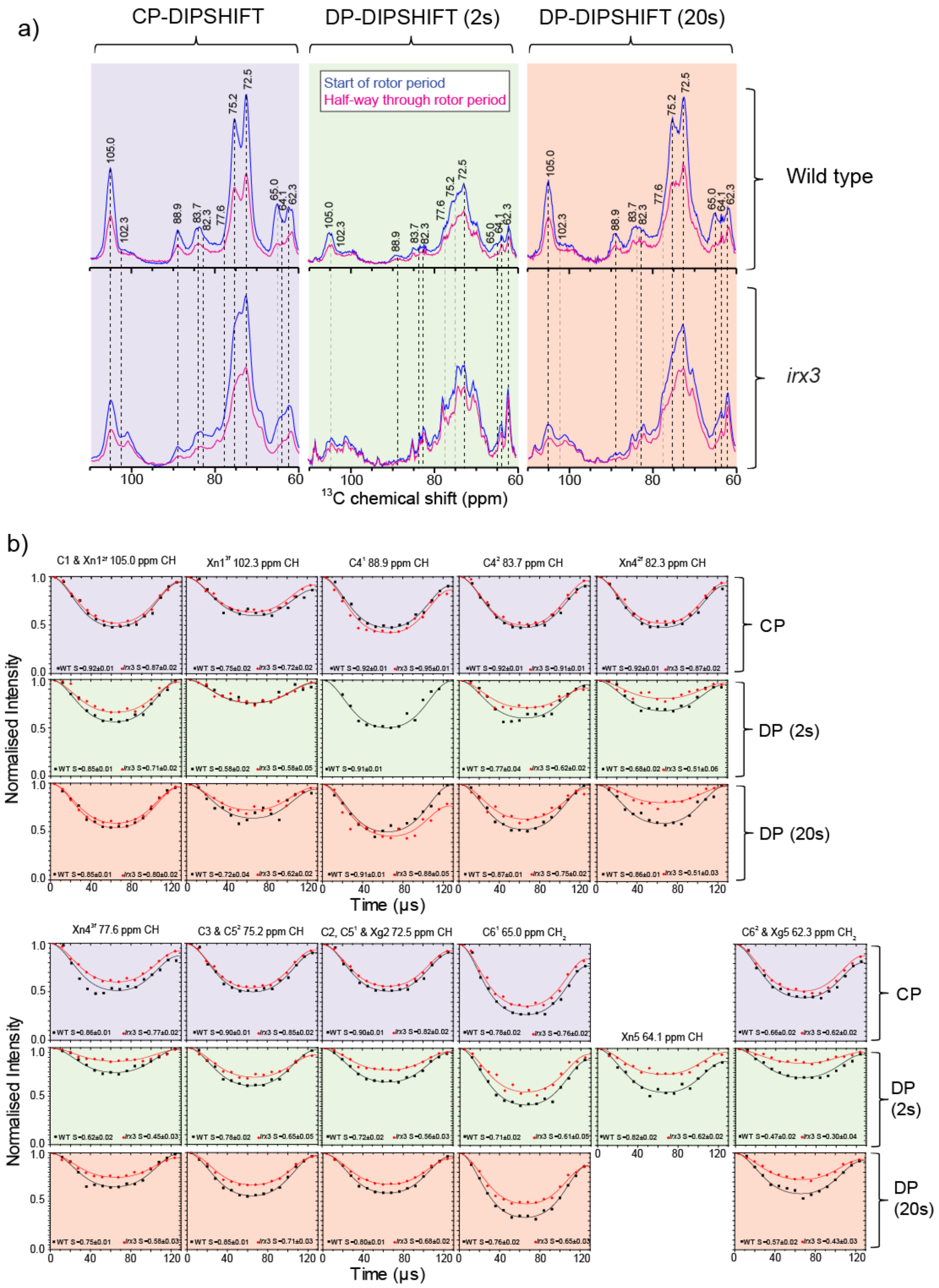

Supplementary Figure 6: DIPSHIFT data for the carbohydrate region in wild type and *irx3*. a, 1D ssNMR spectra showing signals of the carbohydrate region at start (blue) and half-way through (pink) rotor period. **b**, Normalised intensity of selected signals across full rotor period (128 μs) with labels indicating predominant components at these positions; black, wild type; red, *irx3*.

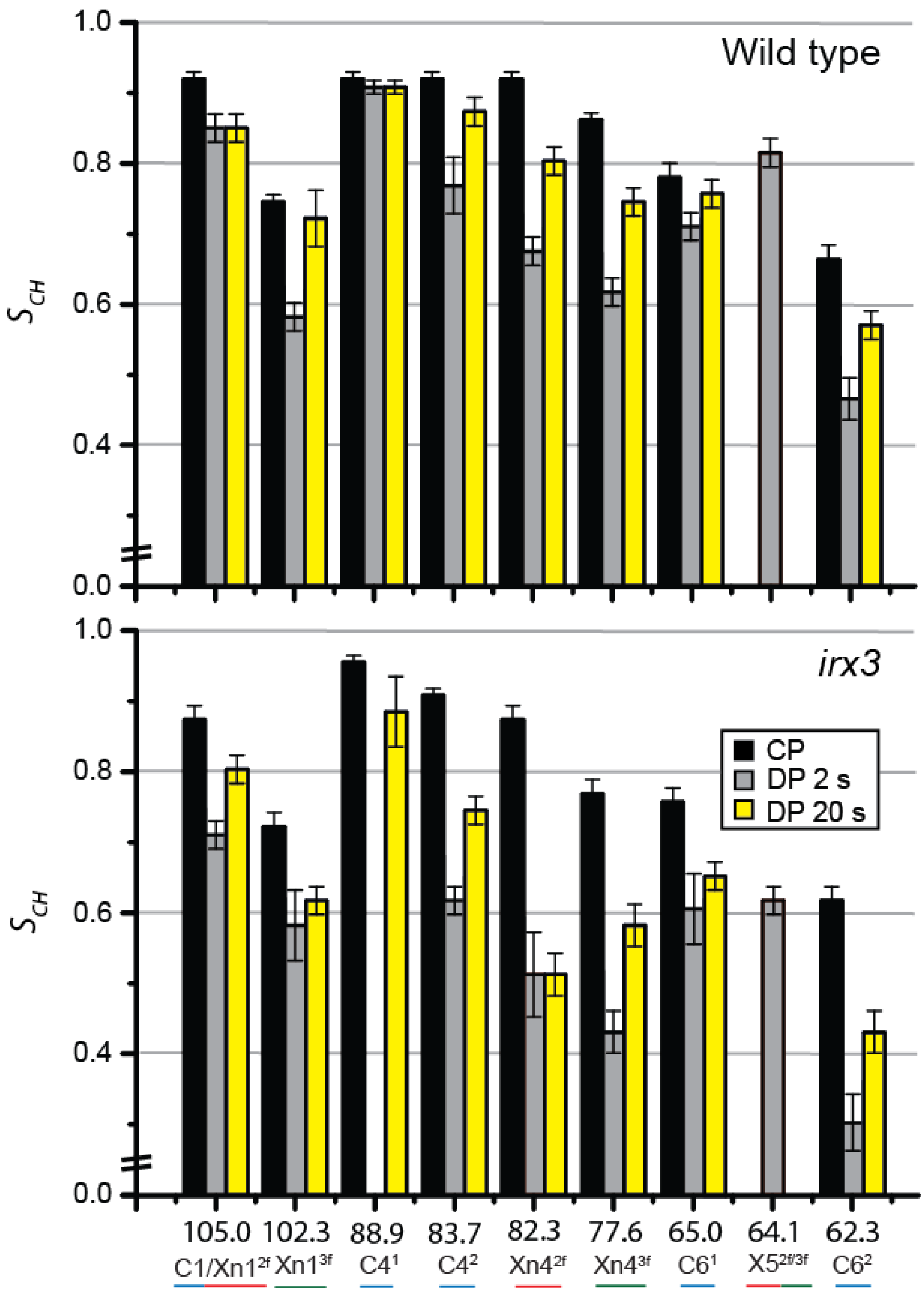

Supplementary Figure 7: DIPSHIFT order parameters for all major signals in wild type and *irx3*. Labels below show major components of the spectra at different chemical shift resonances. See Supplementary Figure 6 for data fits. The error bars were determined by simulation of the DIPSHIFT curves shown in Supplementary Figure 6(b) with varying parameters. The error bar represents the maximum change in parameter before there is a clear deviation of simulated from experimental values.

Supplementary Table 1: ^{13}C NMR Chemical shift values (in ppm) for different moieties.

Polysaccharide or Moiety		Carbon Number						Notes/references
Name	Type, moiety or functionality	1	2	3	4	5	6	
Cellulose	Domain 1 (elsewhere 'interior' or 'crystalline') Glucose	105.0	72.7	75.5	88.8	72.0	65.2	Dick-Perez et al.[3]
		105.1	72.2	74.1	88.6	72.2	64.7	Wang et al.[4]
		**105.1**	**72.8**	**75.7**	**88.9**	**72.7**	**65.1**	Identified in 1D, CP refocussed INADEQUATE
	Domain 2 (elsewhere 'surface' or 'amorphous') Glucose	104.1	72.2	75.0	87.9	70.8	65.6	Wang et al.[4]
		**105.1**	**72.8**	**75.7**	**84.7**	**75.7**	**62.6**	Identified in 1D, CP refocussed INADEQUATE
		**105.1**	**72.8**	**75.7**	**83.8**	**75.7**	**61.7**	Identified in 1D, CP refocussed INADEQUATE
Xylan	3-fold screw Xylose	102.9	73.5	74.3	77.3	63.7	n/a	Averaged shifts of non-modified xylose residues in soluble, extracted *Arabidopsis* xylan[5,6].
		**102.6**	**73.7**	**74.7**	**77.4**	**63.9**	**n/a**	Identified in refocussed INADEQUATE
	2-fold screw Xylose	-	-	-	~81.7*<br>~83.5#	-	n/a	Peak intensity in 1D spectra correlated with xylan prevalence in lignocelluloses[7-9] .
		**105.2**	**72.3**	**(75.2)**	**82.2**	**64.3**	**n/a**	Identified in refocussed INADEQUATE, PDSD
Homogalacturonan	GalA	101.0	69.0	72.0	80.0	71.4	171.3	Dick-Perez et al.[3]
		**100.8**	**68.6**	**68.7**	**79.4**	**71.4**	**171.5**	Identified in CP refocussed INADEQUATE
	Me-GalA	-	-	-	-	-	~173.2	Wang et al.[4]
		**n/d**	**n/d**	**n/d**	**n/d**	**72.8**	**176.1**	Identified in refocussed INADEQUATE
	Me-GalA	~56	n/a	n/a	n/a	n/a	n/a	Hatcher[10]; Holtman et al.[11]
		**53.6**	**n/a**	**n/a**	**n/a**	**n/a**	**n/a**	Identified in CP PDSD
Xyloglucan	Xylose	99.7	72.3	73.8	70.3	62.3	n/a	Dick-Perez et al.[3]
		**99.8**	**72.7**	**74.1**	**70.7**	**62.6**	**n/a**	Identified in CP refocussed INADEQUATE
Acetate	n/a	~174	~21	n/a	n/a	n/a	n/a	Wang et al.[4]
		**173.6**	**21.6**	**n/a**	**n/a**	**n/a**	**n/a**	Identified in CP refocussed INADEQUATE, PDSD

Figures in black are literature assignments shown for comparison. Figures in blue are assignments from the present work.

*Speculated to be "cellulose-aggregated xylan" at accessible fibril surfaces. Not assigned to a specific carbon though recognised as residing in the carbon-4 region.

Speculated to be "cellulose-aggregated xylan" partially composed of xylan at inaccessible fibril surfaces. Not assigned to a specific carbon though recognised as residing in the carbon-4 region

.

Supplementary Table 2: Calculated ^{13}C NMR chemical shifts of 2-fold and 3-fold xylan structures and their difference together with the shift of xylan in solution.

Calculated 2 fold (ppm)	Calculated 3 fold (ppm)	Experimental. Xylan solution (ppm)	Calculated difference 2 fold – 3 fold (ppm)
109.8	105.8	102.5	4.0
72.1	75	73.5	–2.9
73.6	72.3	74.4	1.3
85.9	77.7	77.1	8.2
61.6	60.4	63.7	1.2

Before the shielding calculations, the 10 molecule 2 and 3-fold MD generated xylan structures[5] were first geometry optimised using the CASTEP code[12] with a cutoff energy of 800 ev, a *k* point grid of 2x2x1 and a fixed unit cell of 10x10x60 Å. The NMR shielding was calculated for the resulting structures using the GIPAW[13,14] method. For the 2-fold xylan structure, the shielding of the carbon atoms in adjacent molecules differs by ~<0.1 ppm for the central residues. The differences are somewhat larger for the 3-fold structure being 0.4-0.8 ppm for the two repeat units from residues 3-8 and a mean value was taken. The chemical shift δ is given by $\delta = \sigma_{ref} - \sigma$ where σ is the shielding and σ_{ref} was taken as 167.7 ppm which gives the best fit to the measured solution xylan (non-acetylated *gux*) shifts[5]. Whilst this value of σ_{ref} was determined by assuming that the 3-fold structure is very similar to that of xylan in solution, it is very comparable to that determined for ^{13}C in other organic systems and its value does not affect the calculated shift difference. The geometry optimisation took about 28 hours and the shielding calculations a further 6 hours on Minerva, a regional HPC facility based in the Centre for Scientific Computing at the University of Warwick.

Supplementary Table 3: ^{13}C Spin lattice relaxation time, T_1, at 125.8 MHz Larmor frequency for different chemical shift values.

	WT	WT	*irx3*	*irx3*
Group (^{13}C chemical shift)	T_1 / s [1] (%)	T_1 / s [2] (%)	T_1 / s [1] (%)	T_1 / s [2] (%)
$C_1^{1,2}$, Gal^a, Xn_1^{2f} (105 ppm)		4.5±0.1 (100%)		3.0±0.1 (100%)
Xn_1^{3f} (102.5 ppm)	0.5±0.1 (30%)	4.5±0.3 (70%)	0.34±0.05 (33%)	3.3±0.2 (67%)
C_4^1 (88 ppm)		4.9±0.1 (100%)		4.5±0.3 (100%)
C_4^2, Ara (84 ppm)		3.9±0.2 (100%)		2.9±0.2 (100%)
Xn_4^{2f} , Ara, (82.5 ppm)	0.3±0.1 (20%)	4.3±0.1 (80%)	0.19±0.02 (45%)	2.8±0.1 (55%)
multiple (75 ppm)		4.0±0.1 (100%)	0.18±0.01 (35%)	3.1±0.1 (65%)
multiple (72.5 ppm)	3.0±0.1 (30%)	4.1±0.1 (70%)	0.18±0.01 (25%)	3.3±0.1 (75%)
C_6^1 (65 ppm)		4.3±0.1 (100%)	0.45±0.05 (45%)	5.0±0.3 (55%)
C_6^2, Ara (62 ppm)	0.5±0.1 (25%)	4.4±0.3 (75%)	0.13±0.01 (50%)	2.7±0.1 (50%)

Supplementary References

1. Chen, W. P. *et al.* An automated growth enclosure for metabolic labeling of *Arabidopsis thaliana* with ^{13}C-carbon dioxide - an *in vivo* labeling system for proteomics and metabolomics research. *Proteome Sci* **9**, 9 (2011).

2. Wang, T., Salazar, A., Zabotina, O. A. & Hong, M. Structure and Dynamics of Brachypodium Primary Cell Wall Polysaccharides from Two-Dimensional ^{13}C Solid-State Nuclear Magnetic Resonance Spectroscopy. *Biochemistry-US* **53**, 2840-2854, (2014).

3. Dick-Pérez, M., *et al.* Structure and interactions of plant cell-wall polysaccharides by two- and three-dimensional magic-angle-spinning solid-state NMR. *Biochemistry* **50**, 989-1000 (2011).

4. Wang, T., Zabotina, O. & Hong, M. Pectin-cellulose interactions in the Arabidopsis primary cell wall from two-dimensional magic-angle-spinning solid-state nuclear magnetic resonance. *Biochemistry* **51**, 9846-56 (2012).

5. Busse-Wicher, M. *et al.* The pattern of xylan acetylation suggests xylan may interact with cellulose microfibrils as a twofold helical screw in the secondary plant cell wall of Arabidopsis thaliana. *Plant J.* **79**, 492-506 (2014).

6. Chong, S.L., *et al.* O-acetylation of glucuronoxylan in *Arabidopsis thaliana* wild type and its change in xylan biosynthesis mutants. Glycobiology **24**, 494- 506 (2014).

7. Wickholm, K., Larsson, P. T. & Iversen, T. Assignment of non-crystalline forms in cellulose I by CP/MAS ^{13}C NMR spectroscopy. *Carbohyd. Res.* **312**, 123-129 (1998).

8. Larsson, P. T., Hult, E. L., Wickholm, K., Pettersson, E. & Iversen, T. CP/MAS ^{13}C-NMR spectroscopy applied to structure and interaction studies on cellulose I. *Solid State Nucl. Mag.* **15**, 31-40 (1999).

9. Teleman, A., Larsson, P. T. & Iversen, T. On the accessibility and structure of xylan in birch kraft pulp. *Cellulose* **8**, 209-215 (2001).

10. Hatcher, P. G. Chemical Structural Studies of Natural Lignin by Dipolar Dephasing Solid-State ^{13}C Nuclear-Magnetic-Resonance *Org. Geochem.* **11**, 31– 39 (1987).

11. Holtman, K. M., *et al.* Chemical Structure and Heterogeneity Differences of Two Lignins from Loblolly Pine As Investigated by Advanced Solid-State NMR Spectroscopy *J. Agric. Food Chem.* **58**, 9882– 9892 (2010).

12. Clark, S.J., *et al.* First principles methods using CASTEP. *Z. Kristallogr.* **220**, 567–570 (2005).

13. Pickard, C.J. & Mauri, F. Phys. All-electron magnetic response with pseudopotentials: NMR chemical shifts Rev. B, 63 (2001).

14. Yates, J.R., Pickard, C.J. & Mauri, F. Calculation of NMR chemical shifts for extended systems using ultrasoft pseudopotentials. *Phys. Rev. B* **76**, 024401 (2007).
