# Supplementary material for: Folding of xylan onto cellulose fibrils in plant cell walls revealed by solid-state NMR
Source: Nat Commun. 2016 Dec 21;7:13902. doi: 10.1038/ncomms13902 (PMC5187587; doi:10.1038/ncomms13902)
Supplement: Supplementary Information — Peer Review File [file ncomms13902-s2.pdf]

Reviewers' comments:

Reviewer #1 (Remarks to the Author):

This is an excellent manuscript reporting direct molecular evidence of interactions between xylan and cellulose in intact secondary cell walls of plants. The work is significant and novel, as it demonstrates that solid-state NMR is uniquely capable of revealing the interactions among different plant cell wall polysaccharides as well as the conformation of these polysaccharides. Such native and insoluble plant cell wall materials are impossible to characterize at the molecular structure level by any other physical techniques, the work has high impact to the plant biochemistry and bioenergy fields. Specifically, the authors assigned and resolved two sets of xylan 13C chemical shifts using 2D MAS NMR techniques, and showed that one set of signals has similar chemical shifts as those found in solution and is associated with highly dynamic xylan segments, while a second set of signals has different 13C chemical shifts, comes from rigid segments that interact with cellulose. This xylan-cellulose interaction is observed as cross peaks in 2D 13C correlation spectra. From these data, the authors conclude that while xylan adopts a 3-fold helical screw conformation in solution, in the cell wall xylan changes into a 2-fold helical screw conformation to bind to cellulose. Overall, the data is of high quality, and the conclusion is sound. I recommend publication, after the authors address the following questions to clarify and strengthen their analysis:

1) They should estimate the relative percentages of the 2-fold and 3-fold screw conformations of xylan in their cell walls using the quantitative 13C spectra they already measured. In addition, it will be important to estimate the relative mass of xylan and cellulose in these cell walls. The latter information will be illuminating for understanding how much of the cellulose surface has xylan associated with it.

2) Since it is not possible at present to distinguish the hydrophobic and hydrophilic cellulose signals by NMR, the authors should revise some of the statements in the text and SI figures to make it clear that it remains to be determined whether it is the hydrophilic surface or any surface of cellulose that interacts with xylan. If the quantitative analysis for question #1 gives a xylan coverage of the cellulose surface that is close to the percentage of the hydrophilic surface of cellulose, assuming that this is even known, then this hypothesis could be strengthened.

3) How much of the Arabidopsis stem consists of secondary cell wall and how much is primary cell wall? The authors should comment on how homogeneous or heterogeneous the wall composition is in these samples. Do the five replicates of samples indicated in the SI give similar 2D spectra? Or did the authors add up the spectra from these five samples to give the reported spectra in the figures?

Reviewer #2 (Remarks to the Author):

The major claims of this manuscript is that xylan in fresh Arabidopsis stems adopts a 2-fold screw xylan conformation coating of the hydrophilic faces of cellulose microfibrils and cause surface amorphous cellulose to behave as crystalline cellulose, extending the crystalline core size. This is enabled with 13C labeled Arabidopsis stems that are never dried and confirmed against a cellulose-deficient irx3 mutant.

This work is novel, only a hand full of research groups in the world have the ability to apply solid state NMR in such a detailed fashion. This work will have interest to not only those in the plant biosynthesis world but to those in the biomass conversion community interested to designing plants and processes that facilitates increase cellulase access to cellulose.

This conclusion made in this manuscript are strengthen the conclusions research by other previously most of which was cited.

I don’t have a suggestion: To report monomer sugar distribution and lignin content data for the WT and irx3 mutant. I’m particularly interest in the role lignin might play.

Can you offer an explanation for the range of shifts observed for Xn4 in dried Arabidopsis stems? Are you suggesting that most of the xylan in the WT Arabidopsis stem is bound to the surface of cellulose? If not, is there information in the NMR result to suggest how much of the xylan is this surface bound species. Any evidence of covalently linked hemicellulose and cellulose?

Reviewer #3 (Remarks to the Author):

Review of “Folding of xylan onto cellulose fibrils in plant cell walls revealed by solid-state NMR” by Simmons - Dupree

September 8th, 2016

This is a beautifully written and compelling study and analysis of a problem that has long been poorly understood – that of xylan’s binding to, or interaction with, cellulose. This group has pioneered the remarkable foundational work showing the regularity of xylose substitution in xylans that make this next structural consideration possible. The paper is absolutely of the highest interest to plant cell wall researchers, and has considerable interest beyond just that group.

The combination of MAS NMR to delineate interactions, modeling of course, and the judicious use of a key cellulose-deficient mutant makes this study about as sound as it gets. The group also appreciates the value of using never-dried cell walls, and has done the optimal 13C-labeling experiments to allow the acquisition-time-appropriate use of INADEQUATE experiments.
I confess to not being able to appreciate all the details, but the determinations of relative mobility also fit with the hypothesis for the cellulose binding.

In total, the impressive data presented here from clearly designed experiments allows confidence that the model cautiously proposed by this group is highly likely – to the extent that the hypotheses here, of carefully controlled xylan substitution patterns, and the consequent binding it allows to cellulose (with the flip to the 2-fold screw axis that can be energetically favored during such an interaction), is likely to rather quickly become a full-blown theory.

I had the tiniest of editorial suggestions, but this paper is so well written and obviously carefully edited, that I hardly feel comfortable bringing them up. That said, examples are required (of how trivial they are!)….
1. The use of 'Since' should be restricted to temporal comparisons. There 'Since' in p3-l46 should therefore be changed to 'As'. Ditto for its use on p4-l29 and p6-l19.
2. The 'L' in L-alanine in the experimental is not always reduced 2 point sizes as it should be; e.g., p7-l38. Check throughout.
3. p7-l24: 2 and 3-fold > 2- and 3-fold
4. Units are not always spaced: e.g. in p7-l41: 1ms > 1 ms
5. On p8-l3, the 2T spin-echo > 2tau (sorry, the Greek character as in the spin-echo definition in brackets.
6. The longer en-dash should be used in, e.g., 13C-13C (p8-l8, and ditto for 1H-13C (I thought I saw one that had a normal hyphen, but the en-dash is used correctly on p7-l40). Ditto for C-H in the DIPSHIFT paragraph on p8 and throughout.

The figures are excellent and make good use of color to help the reader, with the exception that the GalA contours are not that easy to see – perhaps they are just made grey to deemphasize them a bit here, in which case that is OK. Figure 2 of the WT vs irx3 mutant is subtle but compelling. Figure 3 is particularly compelling. [B.t.w. The f in 3-fold should be capitalized at the beginning of the sentence!]. Ditto (both points) for Figure 4.

Absolutely one of the most outstanding papers I have been privileged to review this year. Recommendation: Accept essentially as is.

>We are pleased that all three reviewers recognise the novelty and significance of the work, as well as the technical developments needed to make this substantial advance. The work opens a new field with many new interesting questions relating to the way xylan and cellulose interact. Many of the reviewers' questions are important, and will be the topic of experimentation by us and other groups in the years to come.

Reviewer #1 (Remarks to the Author):

This is an excellent manuscript reporting direct molecular evidence of interactions between xylan and cellulose in intact secondary cell walls of plants. The work is significant and novel, as it demonstrates that solid-state NMR is uniquely capable of revealing the interactions among different plant cell wall polysaccharides as well as the conformation of these polysaccharides. Such native and insoluble plant cell wall materials are impossible to characterize at the molecular structure level by any other physical techniques, the work has high impact to the plant biochemistry and bioenergy fields. Specifically, the authors assigned and resolved two sets of xylan 13C chemical shifts using 2D MAS NMR techniques, and showed that one set of signals has similar chemical shifts as those found in solution and is associated with highly dynamic xylan segments, while a second set of signals has different 13C chemical shifts, comes from rigid segments that interact with cellulose. This xylan-cellulose interaction is observed as cross peaks in 2D 13C correlation spectra. From these data, the authors conclude that while xylan adopts a 3-fold helical screw conformation in solution, in the cell wall xylan changes into a 2-fold helical screw conformation to bind to cellulose. Overall, the data is of high quality, and the conclusion is sound. I recommend publication, after the authors address the following questions to clarify and strengthen their analysis:

1) They should estimate the relative percentages of the 2-fold and 3-fold screw conformations of xylan in their cell walls using the quantitative 13C spectra they already measured. In addition, it will be important to estimate the relative mass of xylan and cellulose in these cell walls. The latter information will be illuminating for understanding how much of the cellulose surface has xylan associated with it.

> This is a very interesting question but not easy to answer. To estimate the relative percentages of xylan 2-fold and 3-fold we would need to use quantitative DP spectra (e.g. 20 sec recycle time), but unfortunately the Xn4-2-fold and Xn4-3-fold are not sufficiently resolved from other carbon signals in the 1D spectrum and so cannot be quantitated, - see supplementary Fig. S4c.

Qualitatively, we can say from the CP-INADEQUATE (Figure 1) that the signal of 2-fold is much stronger than 3-fold (revised manuscript page 5, line 133). However, the CP-INADEQUATE 2D spectra are not quantitative, so we prefer not to attempt to provide a numerical measurement for proportions. This is because firstly CP detects only the relatively immobile fraction, and furthermore the INADEQUATE experiment may not be quantitative. However the CP-PDSD (figure 4) likewise shows that in WT the 2-fold xylan signals are much stronger than the 3-fold. For example, the Xn1-2-fold (102.6ppm) cross peak to acetate methyl (21.6ppm) is clearly stronger than Xn1-3-fold (105.2ppm) cross peak to acetate methyl (21.6ppm). These experiments together suggest that the

majority of the immobile xylan is 2-fold. Note also, 3-fold xylan is not detected in the DP INADEQUATE of WT, whereas it is in the *irx3*, suggesting that only a minor fraction of the xylan in wild type is mobile 3-fold xylan (comment added to page 5, line 140).

Together, these data suggest the large majority of xylan in wild type plants is likely to be 2-fold. This is also consistent with the proportion of xylan that is patterned for interaction with xylan according to the hypothesis (Busse Wicher et al. 2016). As requested, we have added a short summary of these results and interpretation at the end of the results section of the manuscript on page 6, lines 213 onwards.

The question of how much of the cellulose surface is another very important point that will take extensive experimentation to address. The plants grown in the $^{13}CO_2$ chamber for these experiments have less secondary cell wall than more robust, older woody plants. Thus, more of the material is primary cell wall than in mature woody tissues, and more of the cellulose is therefore in primary walls without any xylan. Thus ratios of xylan to cellulose measured in this material will not resolve the question. On the other hand, our calculations suggest that in fully mature hardwoods, which contain cellulose:xylan in the ratio 3:2, it is feasible that most of the cellulose surface is coated by xylan. Future experiments will be required to determine this, but we have now added a speculation on page 7 (lines 223-227) that most of the cellulose may be coated in xylan, and certainly there is sufficient to coat the hydrophilic surface. We thank this reviewer for suggesting that interpret the results more fully in these directions.

2) Since it is not possible at present to distinguish the hydrophobic and hydrophilic cellulose signals by NMR, the authors should revise some of the statements in the text and SI figures to make it clear that it remains to be determined whether it is the hydrophilic surface or any surface of cellulose that interacts with xylan. If the quantitative analysis for question #1 gives a xylan coverage of the cellulose surface that is close to the percentage of the hydrophilic surface of cellulose, assuming that this is even known, then this hypothesis could be strengthened.

>The reviewer is correct that any differences in cellulose fibril surface signals are not yet fully interpretable by NMR. Indeed, the surface chains may change their signal once xylan is bound. Therefore, our NMR data do not yet show that it is the hydrophilic or hydrophobic surface or both that is coated by xylan. It is the MD simulations and the evolutionary selection for substitution on alternate residues that lead to the model that it is likely on the hydrophilic faces. We have revised the text for example on page 7, line 220, to clarify this point.

3) How much of the Arabidopsis stem consists of secondary cell wall and how much is primary cell wall? The authors should comment on how homogeneous or heterogeneous the wall composition is in these samples. Do the five replicates of samples indicated in the SI give similar 2D spectra? Or did the authors add up the spectra from these five samples to give the reported spectra in the figures?

> As discussed above, the stems from labelled plants contain a substantial proportion of primary cell wall. We estimate this to be up to one third, based on the proportion of xylan (predominantly

secondary cell wall) and xyloglucan (primary cell wall specific), and is greater in the *irx3* mutant. We have added a comment to this effect in the text on page 9, line 284. This proportion of primary cell wall does not affect the interpretation of the data substantially, since xylan and the *irx3*-dependent cellulose are polymers that are essentially secondary cell wall specific.

The biological replicates, although slightly differing in proportion of primary cell wall, gave similar spectra concerning the xylan shifts. The reported spectra are representative of the results, not averaged spectra.

Reviewer #2 (Remarks to the Author):

The major claims of this manuscript is that xylan in fresh Arabidopsis stems adopts a 2-fold screw xylan conformation coating of the hydrophilic faces of cellulose microfibrils and cause surface amorphous cellulose to behave as crystalline cellulose, extending the crystalline core size. This is enabled with 13C labeled Arabidopsis stems that are never dried and confirmed against a cellulose-deficient irx3 mutant.

This work is novel, only a hand full of research groups in the world have the ability to apply solid state NMR in such a detailed fashion. This work will have interest to not only those in the plant biosynthesis world but to those in the biomass conversion community interested to designing plants and processes that facilitates increase cellulase access to cellulose.

This conclusion made in this manuscript are strengthen the conclusions research by other previously most of which was cited.

I don't have a suggestion: To report monomer sugar distribution and lignin content data for the WT and irx3 mutant. I'm particularly interest in the role lignin might play.

>The sugar content content for the WT and *irx3* mutant are reported in the literature. We have added an additional reference on page 5, line 127. Owing to the small amount of ^{13}C labelled material available for experimentation, we did not repeat these investigations.

The role of lignin is indeed an important question that will require development of further NMR protocols and assignments.

Can you offer an explanation for the range of shifts observed for Xn4 in dried Arabidopsis stems?

>The range of shifts, reported in dried stems, may reflect aggregation of xylan into multiple conformations as reported in Dupree et al. 2015 (ref 28). The effect of drying is not studied in this manuscript and could be the topic of future experiments.

Are you suggesting that most of the xylan in the WT Arabidopsis stem is bound to the surface of cellulose? If not, is there information in the NMR result to suggest how much of the xylan is this surface bound species. Any evidence of covalently linked hemicellulose and cellulose?

>This question is similar to that of reviewer #1. We are indeed suggesting that most of the xylan is bound to cellulose, and have clarified this in the text with the additional paragraph on page 6 and 7 lines 213 onwards. There was no evidence of covalent links, but these would be difficult to detect with these methods.

Reviewer #3 (Remarks to the Author):

Review of “Folding of xylan onto cellulose fibrils in plant cell walls revealed by solid-state NMR” by Simmons - Dupree

September 8th, 2016

This is a beautifully written and compelling study and analysis of a problem that has long been poorly understood – that of xylan’s binding to, or interaction with, cellulose. This group has pioneered the remarkable foundational work showing the regularity of xylose substitution in xylans that make this next structural consideration possible. The paper is absolutely of the highest interest to plant cell wall researchers, and has considerable interest beyond just that group.

The combination of MAS NMR to delineate interactions, modeling of course, and the judicious use of a key cellulose-deficient mutant makes this study about as sound as it gets. The group also appreciates the value of using never-dried cell walls, and has done the optimal 13C-labeling experiments to allow the acquisition-time-appropriate use of INADEQUATE experiments.
I confess to not being able to appreciate all the details, but the determinations of relative mobility also fit with the hypothesis for the cellulose binding.

In total, the impressive data presented here from clearly designed experiments allows confidence that the model cautiously proposed by this group is highly likely – to the extent that the hypotheses here, of carefully controlled xylan substitution patterns, and the consequent binding it allows to cellulose (with the flip to the 2-fold screw axis that can be energetically favored during such an interaction), is likely to rather quickly become a full-blown theory.

I had the tiniest of editorial suggestions, but this paper is so well written and obviously carefully edited, that I hardly feel comfortable bringing them up. That said, examples are required (of how trivial they are!)….

1. The use of ‘Since’ should be restricted to temporal comparisons. There ‘Since’ in p3-l46 should therefore be changed to ‘As’. Ditto for its use on p4-l29 and p6-l19.
2. The ‘L’ in L-alanine in the experimental is not always reduced 2 point sizes as it should be; e.g., p7-l38. Check throughout.
3. p7-l24: 2 and 3-fold > 2- and 3-fold
4. Units are not always spaced: e.g. in p7-l41: 1ms > 1 ms
5. On p8-l3, the 2T spin-echo > 2tau (sorry, the Greek character as in the spin-echo definition in brackets.
6. The longer en-dash should be used in, e.g., 13C-13C (p8-l8, and ditto for 1H-13C (I thought I saw

one that had a normal hyphen, but the en-dash is used correctly on p7-l40). Ditto for C-H in the DIPSHIFT paragraph on p8 and throughout.

We thank the reviewer for the comments and have made editorial changes as suggested.

The figures are excellent and make good use of color to help the reader, with the exception that the GalA contours are not that easy to see – perhaps they are just made grey to deemphasize them a bit here, in which case that is OK. Figure 2 of the WT vs irx3 mutant is subtle but compelling. Figure 3 is particularly compelling. [B.t.w. The f in 3-fold should be capitalized at the beginning of the sentence!]. Ditto (both points) for Figure 4.

We have edited the F in Fold in the figure legends.

Absolutely one of the most outstanding papers I have been privileged to review this year. Recommendation: Accept essentially as is.

REVIEWERS' COMMENTS:

Reviewer #1 (Remarks to the Author):

The authors have addressed my previous questions satisfactorily. However, there are 2 more detailed points that they should correct:

Lines 88-89: “The spectrum shows broad 89 line widths of ~2 ppm indicating the relative rigidity of the polysaccharides”.

This is incorrect. Broad linewidths mean not only rigidity but also static disorder, since a rigid crystalline compound has sharp linewidths.

Line 186-188: “because the Xn42f→C61 is as strong as the Xn42f→Xn52f 187 signal, we can place an upper limit on the distance between the 2-fold screw xylan and cellulose: 2-fold screw xylan can be no farther than the width of a microfibril from the cellulose surface.”

This logic is wrong, which I forgot to include in the initial review. A comparison of the Xn4-C6 cross peak with the intramolecular Xn4-Xn5 cross peak cannot tell the relative Xn-cellulose distances versus the cellulose-cellulose distances. I believe the authors meant to compare the Xn4-C6 cross peak with the C6 domain 1 - domain 2 cross peak. Please clarify.

Reviewer #2 (Remarks to the Author):

The authors responses were clear and satisfactory.

We value the helpful suggestions that Reviewer #1 has made and the manuscript is being improved by the revisions. Our response to these further two suggestions is as follows:

Reviewer:

Lines 88-89: “The spectrum shows broad 89 line widths of ~2 ppm indicating the relative rigidity of the polysaccharides”.

This is incorrect. Broad linewidths mean not only rigidity but also static disorder, since a rigid crystalline compound has sharp linewidths.

Response:

If there were a substantial amount of static disorder the linewidth would be much greater. It is likely that the broad linewidths result from a range of different environments in addition to rigidity. Therefore we suggest a change to the sentence as follows:

“The spectrum shows broad line widths of ~2 ppm indicating the relative rigidity of the polysaccharides and a range of different environments”.

Reviewer: Line 186-188: “because the Xn42f→C61 is as strong as the Xn42f→Xn52f 187 signal, we can place an upper limit on the distance between the 2-fold screw xylan and cellulose: 2-fold screw xylan can be no farther than the width of a microfibril from the cellulose surface.”

This logic is wrong, which I forgot to include in the initial review. A comparison of the Xn4-C6 cross peak with the intramolecular Xn4-Xn5 cross peak cannot tell the relative Xn-cellulose distances versus the cellulose-cellulose distances. I believe the authors meant to compare the Xn4-C6 cross peak with the C6 domain 1 - domain 2 cross peak. Please clarify.

Response:

We have reconsidered this carefully. The comparison of the Xn4-C6 cross peak to the intramolecular Xn4-Xn5 cross peak was to show that the spin diffusion is substantial. Although spin diffusion in a fully 13C labelled system is complex, we feel it is useful to compare the Xn4-C6 cross peak with the intramolecular Xn4-Xn5 cross peak, but, as the reviewer indicates, we should compare both these cross peaks to the C6 domain 1 - domain 2 cross peaks which are not yet as strong at this mixing time. We conclude that the xylan:cellulose distance is less than the distance between the two cellulose domains- which is maximally the width of the fibril. We have modified the wording to make this clearer. The modified wording is below:

“The similar signal strengths of the intermolecular $Xn4^{2f}\rightarrow C6^{1}$ and the intramolecular $Xn4^{2f}\rightarrow Xn5^{2f}$ peaks indicate that almost all of the 2-fold screw xylan is spatially close to cellulose. In contrast at this mixing time the cross peaks showing spatial

proximities between cellulose domains (e.g. $C4^1 \rightarrow C6^2$ and $C6^1 \rightarrow C6^2$) are not yet as strong as those showing spatial proximities within cellulose domains (e.g. $C4^1 \rightarrow C6^1$). Although spin diffusion in a fully ^{13}C labelled system is complex, the presence of the relatively large $Xn4^{2f} \rightarrow C6^1$ cross peak means that we can place an upper limit on the distance between the 2-fold screw xylan and cellulose: 2-fold screw xylan can be no farther than the width of a microfibril from the cellulose surface. Hence, the PDSD experiments indicate that the 2-fold screw xylan is bound to cellulose."

We trust that this suitably clarifies these queries.
